# Aesthetic Rehabilitation Using Various Techniques for Anterior Teeth Affected by Structural Damage Due to Detrimental Habits: A Case Series

**DOI:** 10.7759/cureus.97984

**Published:** 2025-11-27

**Authors:** Navdeep Jethi, Subhankar Banik, Puspita Guha

**Affiliations:** 1 Conservative Dentistry and Endodontics, Daswani Dental College and Research Centre, Kota, IND; 2 Conservative Dentistry and Endodontics, Agartala Government Dental College and IGM (Indira Gandhi Memorial) Hospital, Agartala, IND; 3 Conservative Dentistry and Endodontics, Agartala Government Dental College, Agartala, IND

**Keywords:** dental aesthetics, glass ionomer cement (gic), lithium di silicate, porcelain laminate veneer, post-and-core joint crown, vital pulp therapy

## Abstract

In this case series, various techniques are employed to address non-carious and structural damage to teeth resulting from harmful habits, such as the use of abrasive tooth powders and parafunctional habits such as tearing wires. The initial case involved the use of the hybrid sandwich veneering technique, which provided structural reinforcement and aesthetic enhancement for the upper anterior teeth affected by abrasion. Initially, the buccal surfaces of these teeth underwent treatment with calcium hydroxide, followed by restoring the cavosurface with restorative glass ionomer cement for indirect pulp protection. Subsequently, veneers (E. max) were bonded onto the maxillary central incisors using luting resin-based cement. Also, the fracture of the lower central incisor was addressed through metal post-reinforcement, followed by the placement of a crown. In another case, lithium disilicate (E. Max) veneers were used to enhance aesthetic concerns by addressing incisal corrections and midline issues specifically.

## Introduction

Abrasion is the wearing down of teeth by external materials or substances like abrasive dental powders or toothbrushes [1].

The use of abrasive dentifrices like red tooth powder (known as *Lal Dant Manjan*) and harmful occupational habits can lead to significant dental structure loss [1]. This damage is often due to the presence of irregularly sized iron oxide particles in tooth powders [1]. Abrasive tooth powders can cause non-carious damage like frictional wear, gum irritation, and enamel erosion, leading to structural issues [1,2]. The use of abrasive tooth powder causes friction over the enamel layer, leading to wear, sensory issues, and aesthetic problems in the anterior teeth [3].

Using a hard toothbrush and non-polished, rounded fibers can cause toothbrush abrasion, leading to frictional wear on the teeth. This particularly affects the cervical or incisal parts, resulting in enamel and gum damage as well as increased sensitivity [4]. Parafunctional habits like nail biting, teeth clenching, bruxism, and using teeth to hold objects can contribute to noncarious structural damage in the teeth. Individuals in tailoring and electrical work are at a higher risk of damaging their teeth due to occupational habits, which can result in enamel loss and even tooth fractures [5]. Research data shows a higher prevalence of abrasion and incisal edge wear among tailors and electricians, similar to the increased erosion rates seen in employees of acid factories. This highlights the significant occupational impact on tooth wear [6].

In Case 1, a minimally invasive technique was applied to restore maxillary central incisors facing abrasion and dentine loss due to the prolonged use of red tooth powders containing iron oxide and the habit of using teeth for holding and tearing wires [1,6]. Modified sandwich veneering comprises four layers for restoration: calcium hydroxide for pulp protection, glass ionomer cement for sealing to minimize microleakage, resin cement for luting, and porcelain veneer for aesthetics, specifically used in indirect pulp capping procedures [7,8]. Also, in this case, another issue was detected. The mandibular right lateral incisor, which faces the crown en masse, fractures due to occupational habits such as wire tearing, constituting Case 2 of structural damage due to determinantal habits [9]. This case was managed by a metal post and core followed by porcelain-fused crowns over the mandibular anterior teeth. In Case 3, structural integrity of the incisal edge was restored with lithium disilicate (E. Max) veneers to enhance aesthetic concerns [10].

This case series employs various veneering techniques, customized, precise digital impressions, and computer-aided design (CAD)/computer-aided manufacturing (CAM) technology, offering a conservative approach to enhancing the natural appearance of teeth, effectively addressing aesthetic concerns.

## Case presentation

Case 1

Patient History

A 26-year-old male patient reported to the department of conservative dentistry and endodontics with a complaint of a dislodged restoration and general sensitivity in the upper anterior teeth. The patient had a history of restorations in the upper and lower central incisors three months ago. The patient used abrasive tooth powder (*Lal Dant Manjan*) as a dentifrice, either by finger or with a toothbrush, and practiced vigorous, improper brushing. When asked, he mentioned that the bristles on his toothbrush had worn out within two weeks of use. Also, he had the habit of holding with his anterior teeth, and he used to tear the coating of electric wires with his teeth frequently as an occupational habit.

Intraoral Examination

The patient exhibited vigorous abrasions on the maxillary central incisors, along with a moderate cervical abrasion on the mandibular right central incisor, specifically on the facial surfaces. A pink pulpal spot, which indicates potential pulp involvement and the need for further evaluation, was visible under the sparse layer of dentin in the middle third of the buccal aspect of the crown in teeth 11 and 21. Buccal and incisal surfaces were affected in teeth 11 and 21. In the lower central incisors, cervical abrasion was seen, and the right incisor was cervically restored.

The electric pulp vitality tests confirmed the health of the pulp, and vertical tapping did not elicit tenderness in the teeth, indicating overall dental health.

Treatment Plan

Considering aesthetic concerns, the decision was made to address maxillary teeth first with pulp capping and porcelain laminate veneers using hybrid sandwich veneering (Table 1). Subsequently, the lower teeth were scheduled for cervical restoration. However, an unexpected crown fracture in tooth 41 due to wire tearing led to a revised plan involving metal post-reinforcement and crowns for pain management in teeth 31 and 41.

**Table 1:** New hybrid sandwich veneering with four layers of direct and indirect restoration

	Hybrid Sandwich Veneering
1.	Calcium hydroxide over a pink pulp spot
2.	Glass ionomer cement over calcium hydroxide
3.	Resin cement over the contoured cavosurface margin of the GIC and dentine layer
4.	Veneer/laminate with resin cement

Indirect Pulp Capping

**Figure 1:**
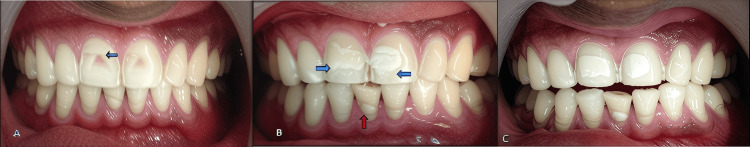
A) Abrasions and pink pulp spots in teeth 11 and 21; B) pulp protection using a calcium hydroxide base under a glass ionomer cement; C) facial preparation in 11 and 21

The pulp in this case was not directly exposed, but pink pulp spots were visible in the middle of the buccal surfaces of the crown in teeth 11 and 21, under a thin layer of dentine. The area was isolated and dried to prevent moisture incorporation, ensuring optimal conditions for the procedure. In this hybrid sandwich veneering technique, the first step was to protect the pulp from irritants. Calcium hydroxide was placed over the pink spot for indirect pulp capping (Table 1). This was followed by the application of restorative glass ionomer cement (GC Gold Label Universal Restorative, GC Corporation, Tokyo, Japan) to contour the tooth's surface before buccal surface preparation (Figures 1A-1C).

Veneer Preparation

After the setting of the glass ionomer cement, the buccal and incisal edges of 11 and 21 were prepared using a taper flat bur. Enamel reduction was approximately 0.4-0.6 mm, required for both strength and aesthetics. A two-faced reduction was done (Figure 1C).

On the lingual surface, the preparation of the incisal edge was extended by 2 mm, involving the proximal contacts to the same length over the incisal edge.

**Figure 2:**
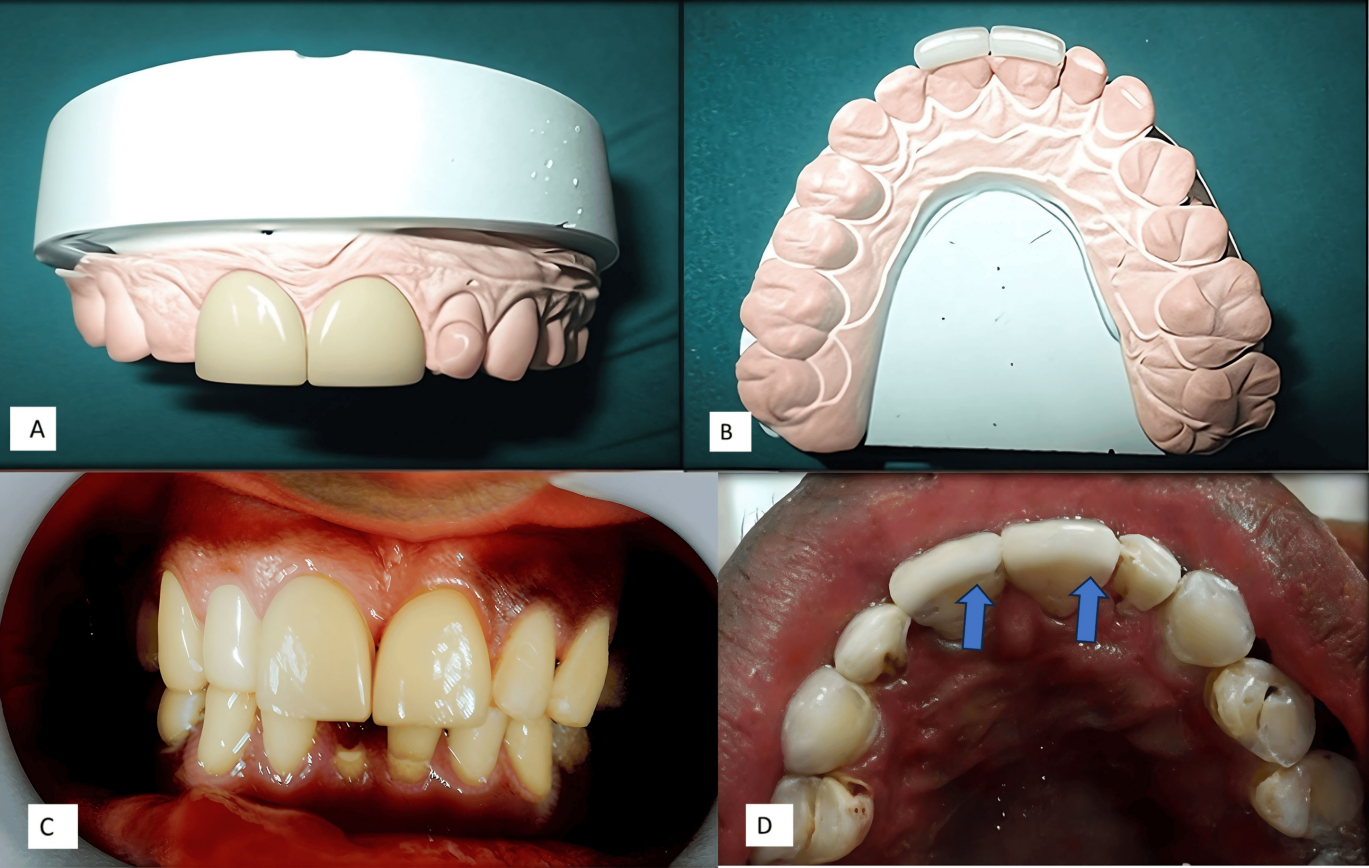
A) facial view of the veneers on cast; B) incisal view of the veneers on cast; C) fracture of tooth 41 and veneer placement (11 and 21) facial view; and D) incisal view after veneer placement

A digital impression was taken for the veneer preparation, and the color was selected using the Vita shade guide and the A1 shade was chosen to match the natural color of the teeth under moist conditions in natural light. A cast was made to facilitate CAD and CAM manufacturing of the veneers by the lab technician (Figures 2A, 2B).

Cementation of Veneers

The next day, both veneers were tried all at once to evaluate the fit and the interproximal contacts. After doctor and patient acceptance, initially, the restorations were cleaned. They were then etched with 35% phosphoric acid, silanated, and set aside. Next, the teeth were treated with phosphoric acid gel for 10 seconds to prepare them for bonding, followed by rinsing. Multiple coats of a fifth-generation bonding agent were applied, lightly air-dried, and then light-cured for 10 seconds per tooth. A light-cured luting resin cement (3M RelyX Ultimate Clicker ) was placed on the internal surface of the restorations, and the restorations were then seated into place. Cotton rolls and brushes were used to clean excess cement from the buccal and lingual surfaces and from interproximal surfaces using a sickle-shaped explorer instrument and a spoon excavated with cotton rolls and brushes. The restorations were then positioned at the gingival margin using a 3-mm curing light tacking tip for 5 seconds per tooth. After removing excess cement interproximally with dental floss, light-curing was done for one minute on each surface (facial and lingual) using a light-emitting diode (LED) curing light (Figures 2C, 2D).

Finally, the restorations were polished using rubber points. The patient was scheduled for a follow-up the next day for occlusal adjustments, and the restorations were assessed for aesthetics, function, and phonetics to ensure proper fit and appearance. The patient's positive feedback, which included minimal discomfort, no chewing or speaking difficulties, and high satisfaction with the feel and appearance of his teeth and smile post-treatment, clearly indicates a successful outcome of the treatment.

Case 2

Another case of structural damage due to detrimental habits was noted in the patient in Case 1.

Crown En Masse Fracture in Mandibular Central Incisor (Tooth 41)

**Figure 3:**
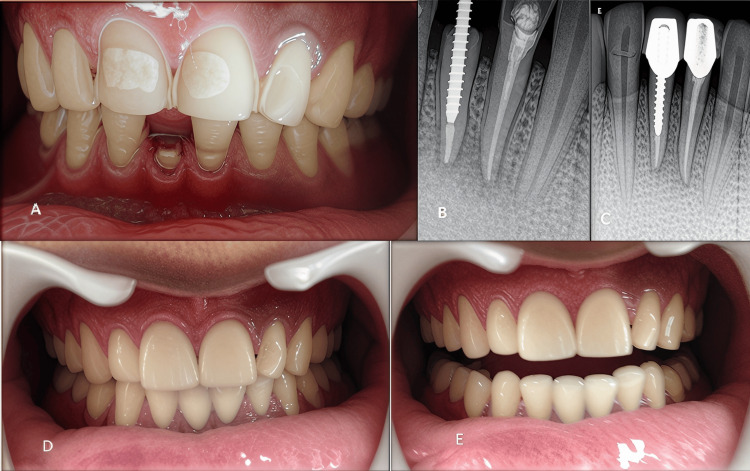
A) fractured tooth 41; B) radiographs for metal posts in 41 and RCT in 31; C) PFM crowns in mandibular teeth; D) postoperative photograph; E) Follow up after two years RCT: root canal treatment; PFM: porcelain fused to metal

Before the cementation appointment, the patient had the crown fractured in 41 and suffered pain in 31 due to trauma caused during the tearing of a wire coating. Both teeth were scheduled for root canal treatment (Figure 3A).

**Figure 4:**
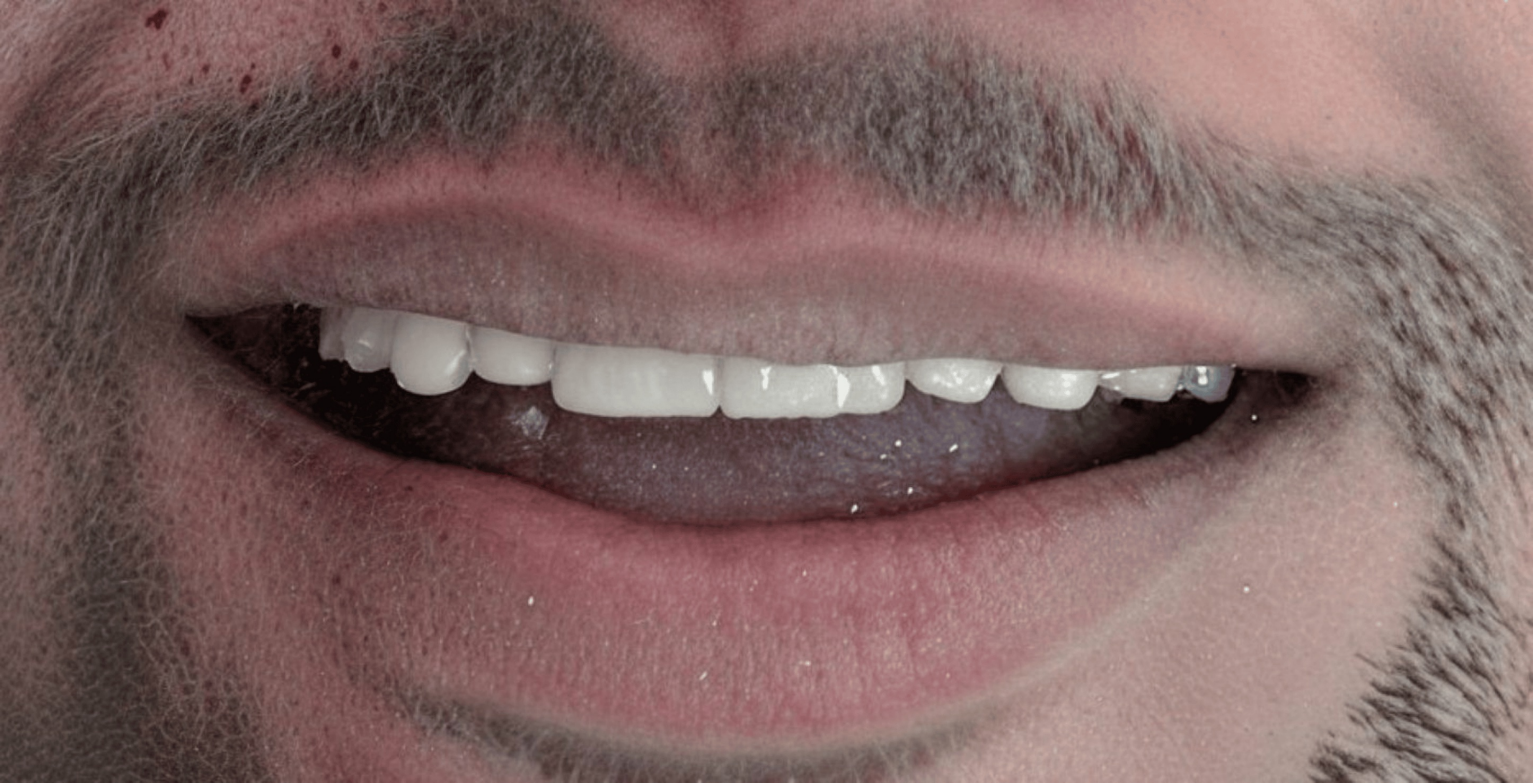
Post veneer smile

To strengthen tooth 41, a metal post was inserted with the post space prepared up to 10 mm, leaving 5 mm of gutta-percha at the apex (Figures 3B, 3C). The metal posts were secured with resin cement, and the tooth structure was restored (core build-up in 41) using composite material (Tokuyama Omnichroma, Tokuyama Dental Corporation, Tokyo, Japan) before porcelain-fused crowns were placed on the mandibular central incisors (Figures 3D-3E and Figure 4).

Dental Instructions

The patient was counseled against detrimental habits like using teeth for tearing wires, biting nails, or holding objects, as these actions can cause severe dental damage, leading to compromised oral health. The patient was educated to discontinue rubbing teeth with abrasive tooth powders during the dental consultation. It was stressed how crucial it is to adopt correct brushing techniques to maintain optimal oral health.

Follow-Up

The patient returned after two years for follow-up appointments (Figure 3E). The veneers and PFM crowns were intact, and no sensitivity or pain was reported.

Case 3

**Figure 5:**
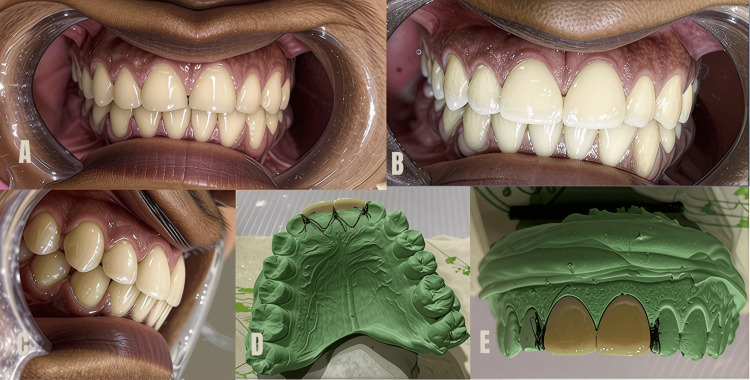
A) Before the application of veneers (teeth 11 and 21), B) and C) after the cementation of veneers, and D) and E) veneers on the cast

A 22-year-old patient visited the conservative dentistry department, expressing concern about wear on their anterior teeth and requesting aesthetic enhancement. The patient's history revealed the habit of nail biting and holding sewing needles and other objects between their anterior teeth, resulting in chipping and flattening of the incisal edges (Figure 5A). Aesthetic veneers were chosen to correct the black triangle between central incisors (11 and 21) and for incisal edge correction (Figures 5B, 5C). The buccal surface and incisal edges of teeth 11 and 21 were prepared using a diamond bur. Enamel reduction was approximately 0.4-0.6 mm, required for both strength and aesthetics. A two-faced reduction was done. On the lingual surface, the preparation of the incisal edge was extended by 1 mm, involving the proximal contacts to the same length over the incisal edge. A digital impression (Helios 600 - 3D Intraoral Scanner, Eighteeth, Jiangsu, China) was taken for the veneer preparation, and the A2 shade from the Vita shade guide was chosen to match the natural color of the teeth under moist conditions in natural light, providing a harmonious aesthetic outcome. A cast was made to facilitate CAD and CAM manufacturing of the lithium disilicate glass-ceramic (E. max) veneers by the lab technician (Figures 5D, 5E).

The cementation procedure was the same as in Case 1, but in Case 3, 3M ESPE RelyX Veneer Cement, known for its strong bonding properties, was used for veneer cementation.

The patient was counselled against detrimental habits like using teeth for holding needles, highlighting the risks of dental damage and potential injuries associated with such habits.

## Discussion

In these cases, detrimental habits like biting on hard objects (Cases 1-3) and the use of abrasive tooth powder led to tooth damage.

In Cases 1 and 2, the patient used abrasive tooth powder as a dentifrice, which significantly contributed to the abrasion and attrition of the central incisors. Research by RP Singh et al. (2016) concluded that the presence of iron oxide (high quantity) in *Lal Dant Manjan* and Baidyanath Tooth Powder causes the wear of the tooth surfaces [1]. Studies by Ahn et al. (2018) and Osmanaj Nhave (2022) have explored the abrasive behavior of specific dentifrices such as charcoal and tooth powders, respectively [2,3]. Their findings concluded that these products can lead to an increase in dentinal hypersensitivities and a higher risk of tooth staining [1-3].

According to a systematic review by Paula et al., the clinical performance of restorations in non-carious lesions using the sandwich technique exhibited a 20% rise in success rates during three-year follow-ups, surpassing cases treated solely with composite resins [7]. In the sandwich technique, glass ionomer cement is utilized as a liner beneath the composite resins to establish a more robust and enduring bond with the dentine and cavosurface margin, enhancing durability and reducing microleakage to the pulp [11].

In contrast, in Case 1, hybrid sandwich veneering was utilized prior to porcelain laminate placement, which involved incorporating indirect pulp capping with traditional calcium hydroxide [12,13]. The placement of the second layer of high-strength glass ionomer aimed to minimize issues such as microleakage, marginal adaptation, and contour below the veneer preparation, and the third layer of luting resin below the veneer [11]. The chemical and adhesive bonding properties of the glass ionomer cement (GIC) create a strong connection with the dentine and facilitate the adhesion of resin cement (3M ESPE Ultima Clicker) used for bonding the porcelain laminates, ensuring reliable adhesion. The fourth layer of laminate veneer completes the four-layer hybrid sandwich of restoration [8].

Hybrid sandwich veneering not only preserves the current tooth structure but also plays a crucial role in safeguarding pulp tissues and the natural blood supply to teeth. This process helps prevent discoloration of the tooth beneath porcelain laminate veneers (PLVs) by maintaining the natural color and appearance of the tooth [8,11].

In Case 3, the incisal edge was flattened due to the detrimental habit of holding a needle in between the teeth and nail biting, and in Case 1, the use of anterior teeth for tearing off wire coating was reported. Emphasizing the importance of incisal preparation is crucial to reinforce the vulnerable incisal edge of the tooth, protect it from damage caused by biting on hard objects, and ensure long-term durability [14]. This can potentially lead to chipping or fractures, especially in cases of non-carious tooth loss due to occupational habits [5]. Avoid habits such as biting on wire coatings, nails, and hard objects to prevent veneer dislodgement and breakage, ensuring the long-term durability of your dental restorations [3,5]. These habits can compromise dental restoration by increasing the risk of damage, instability, and the potential failure of the restorative work [2,5].

A two-unit bridge was planned for the mandibular central incisors (Case 2) to restore functionality and provide structural support after substantial tooth loss resulting from the crown-en-mass fracture, ensuring stability in the affected region [15]. In accordance with previous literature, the aesthetic outcomes were more natural in the maxillary teeth with porcelain laminate veneers due to their translucency and lifelike appearance compared to the mandibular teeth with PFM crowns. In cases where porcelain-fused crowns chip off, they expose the underlying silver color of the metal [16,17]. This situation can give rise to aesthetic concerns and jeopardize the natural appearance of the teeth, particularly in the visible anterior region, impacting the overall smile aesthetics and patient satisfaction [18,19]. In Case 3, lithium disilicate veneers (E.max) were utilized because they offer greater strength, superior aesthetics, and enhanced durability compared to traditional porcelain veneers [10]. This allows for thinner and more minimally invasive preparations [10]. Zirconia generally offers superior mechanical strength and fracture resistance, making it ideal for high-stress areas, while lithium disilicate is often preferred for its higher translucency and superior aesthetics, especially in the anterior region [20].

## Conclusions

Veneers are an important modality to address aesthetic problems in anterior teeth. Abrasive dentifrices and occupational habits in certain professionals are the primary factors contributing to non-carious structural tooth loss. These practices can lead to dentinal sensitivity or pulp damage in the long term, and the associated aesthetic problems can be financially burdensome. A hybrid sandwich veneering is a minimally invasive conservative technique that can prevent pulp damage in cases of attrition and abrasion and offers superior aesthetics over porcelain-fused metal crowns. Lithium disilicate veneers can provide more durability and aesthetics than porcelain laminate veneers.
